# Turning over New Leaves

**Published:** 1890-04-26

**Authors:** 


### Turning Over New Leaves.

**Dr. H. Kuhne's Guide to the Demonstration of Bacteria in the Tissues.***

This small work will prove of great service to those unacquainted with the technique of bacteria staining. The differential methods of staining tissues containing micro-organisms are well described, and anyone who carefully follows the instructions given can hardly fail to obtain the most satisfactory results. The methods of making dry cover-glass preparations, together with the staining of tubercle bacilli with carbol-fuchsin are given in detail, and are most valuable. Dr. Kühne has only described the *freezing* method of cutting sections, but in the appendix the translator has added an account of the celloidin and paraffin methods, which adds greatly to the merit of the work. The celloidin method is of great value in preparing sections of delicate tissues, such as spleen, or spinal cord, which cannot be satisfactorily frozen without injury. He has also wisely added an account of other microtomes, besides the freezing one of Katsch, but gives little more than a list of makers. We ourselves prefer Reichert's microtome (which can be used for both celloidin and freezing) to any other, for it serves all purposes, and is decidedly the most inexpensive. The appendix of formulæ will be found useful for reference, but in formula I. there is apparently a misprint; a 5 per cent., and not a 50 per cent. solution of carbolic acid should be used.

In reference to the manner in which the translation has been performed, we are sorry not to be able to accord to it the same approval we have given to the material. It has apparently been somewhat hastily put together, and has been rendered too literally from the German. It is consequently a little difficult to comprehend. But in spite of this defect we can confidently recommend the book to those interested in bacteria, and the methods of demonstrating them in animal tissues. It contains a large amount of valuable material in a small compass, and as a laboratory companion it is one of the most useful works of the kind with which we are acquainted.

* "Dr. Kuhne's Guide to the Demonstration of Bacteria in the Tissues." Translated by Dr. N. D. Harris. (London: Balliere, Tindall, and Co.)

**Egypt as a Winter Resort.***

This is an extremely valuable little book, and one that may be recommended not only to invalids but to all who intend visiting the land of Egypt. As might be expected from the name of the author, it stands on a far higher platform than the majority of such works. There is nothing of the showman about it.

While telling us of the advantages of a residence in Cairo, Mr. Sandwith does not conceal the drawbacks, and we can hardly do better than transcribe, with some condensation, what he says on these points. The advantages are a dry and exhilarating air; an almost complete absence of rain; the absence of extreme cold; the absence of strong winds; the interest and relaxation of mind inspired in a country where the remains of ancient Egyptians, Persians, &c., may be studied; the presence of European luxuries; the absence of moribund phthisical patients, and the facilities for riding and driving. The objections are its distance from England; the expenses which attend the journey; the mosquitoes; a certain monotony at the dinner-table of butcher's meat, poultry, and game; and the insanitary condition of the picturesque old parts of Cairo. Undoubtedly, the advantages far outweigh the objections, and when we read the descriptions of the cloudless skies and the moonlit evenings on the desert; we could almost wish our physician would order us to Egypt if we were not very ill, for health is before everything. Then there is the grand Egyptian architecture associated with the dreams of our youth, and with the studies of our maturity. Everything conspires to insist on the invalid forgetting his troubles; the mind having so much else to dwell on abandons introspection, and bodily elasticity takes the place of mental lassitude.

There are several tables in the book showing the average temperature, the rainfall, the force of the wind, &c., during the various months and seasons. At the end is an appendix with short notes on the diseases which are cured or modified by a residence in Egypt.

**A Manchester Shirt Maker.***

Since Tom Hood wrote "The Song of the Shirt," many a writer has in verse and prose bewailed the hard lot of the poor seamstress. Mr. Law, in "A Manchester Shirt Maker," tells us the old story once more; and if there be nothing strikingly original in the plot; nothing very good in the portrayal of the characters; if the situations be decidedly improbable, we may pardon all these defects in consideration of the object with which the story was written. There are plenty of men in England who would gladly pay a shilling or two a-dozen extra for their shirts could they but feel sure that the money would find its way into the pockets of the makers, and not into those of the middlemen or retailers. Cannot Mr. Law or someone else tell us how it could be done? If the system of employing middlemen such as described in this little book, or even middlemen of any kind, be common among our shopkeepers, we can only say that it is an eternal disgrace to the heads of these establishments, who must be well aware of what the general public cannot know. Mr. Law makes his shirtmaker murder her child, and then escape execution on the ground of insanity. Why she was sent to the County Asylum near Manchester, and not to Broadmoor, he does not tell us; and we hope the present assistant medical officers at the County Asylum are neither fortune-hunters nor Socialists, as, according to Mr. Law, they were at one time.

* "A Manchester Shirt Maker." By John Law. (Co-operative Publishing Company, London.) Pp. 168.) Price 1s.

**Influenza and Colds.***

We confess to preferring the popular to the pedantic style, but Dr. Fernie pleases us overmuch. On page 43 of his book on influenza, for instance, he begins, "By long chalks, as the saying runs, the best way to escape the epidemic is to religiously avoid reading about it in the daily papers, etc." The conclusion of the same page is as follows: "When a rose-coloured rash makes its appearance simultaneously with high temperature, and with catarrhal flux from the nose and eyes, as well as with frontal headache, it will be found useful to administer small doses of arsenic in alternation with the aconite or the green hellebore, as the case may be." Now is this addressed to the populace or to the profession? If to the populace, then by his showing Dr. Fernie is inviting them to take influenza by inviting them to read about it; if to the profession, then, as we said before, the style is too popular even for us. The fact is that a book rushed through the press to meet a temporary want ought not to be reviewed—it ought only to be advertised. Dr. Fernie is a great believer in the old-fashioned onion porridge for a cold in the head, and the homœopathic principle that if the onion causes watering of the eyes, sneezing, etc., when taken in harmful quantities, modified forms of the onion are likely to have a curative effect when similar morbid symptoms arise from other causes. This book is brightly written, and full of useful hints on minor matters; let us hope it fulfilled its mission while the epidemic lasted.

* "Influenza and Colds." By W. T. Fernie. (Percival and Co.) Price 2s.

